# The role of renal damage markers in the diagnosis of early stages of kidney injury in patients with latent autoimmune diabetes in adults

**DOI:** 10.25122/jml-2022-0062

**Published:** 2022-06

**Authors:** Iryna Tsaryk, Nataliia Pashkovska

**Affiliations:** 1 Department of Clinical Immunology, Allergology and Endocrinology, Bukovinian State Medical University, Chernivtsi, Ukraine

**Keywords:** diabetes mellitus, latent autoimmune diabetes in adults, cystatin C, creatinine, glomerular filtration rate, chronic kidney disease, A+ – albuminuria, A- – normoalbuminuria, ADA – American diabetes association, antiGAD – antibodies to glutamic acid decarboxylase, CKD – chronic kidney disease, DKD – diabetic kidney disease, DM – diabetes mellitus, DN – diabetic nephropathy, GFR – glomerular filtration rate, LADA – latent autoimmune diabetes in adults, MAU – microalbuminuria, T1DM – type 1 diabetes mellitus, T2DM – type 2 diabetes mellitus

## Abstract

Serum creatinine level begins to increase after a decrease in glomerular filtration rate (GFR) by 50% and more, so the question emerged about a more accurate method of determining GFR. The study aimed to determine the role of renal damage markers in the diagnosis of early-stage renal disease in patients with latent autoimmune diabetes in adults (LADA). We included 84 patients with diabetes mellitus (DM) and chronic kidney disease (CKD) caused by diabetic kidney disease (DKD), as well as 25 representatives of the control group. Patients were divided into three groups – 43 people with LADA, 21 with type 1 diabetes mellitus (T1DM), and 20 patients with type 2 diabetes mellitus (T2DM). GFR was assessed using six formulas after establishing the category of GFR and albuminuria. The GFR rate estimated by the CKD-EPI formula in patients with LADA and DKD did not significantly differ from that of CKD-EPI cysC, slightly different from MDRD GFR (10.6% higher, respectively) but 21.9% lower compared to CG formula. In patients with LADA and T1DM, GFR was higher in cases with existing albuminuria, regardless of the formulas used. Thus, the non albuminuria phenotype is accompanied by a greater degree of renal impairment, which indicates the need to determine serum cystatin C in the early stages of LADA. Cystatin C levels are the most accurate, early, and independent predictor of the development and progression of CKD in patients with DM, including LADA.

## INTRODUCTION

Diabetic nephropathy (DN) is one of the main chronic microvascular complications in patients with diabetes mellitus (DM) and the leading cause of end-stage chronic kidney disease (CKD) [1]. In 2014, the American Diabetes Association (ADA), together with the National Kidney Foundation (NKF), recalled DN and CKD caused by diabetes with a constant assessment of glomerular filtration rate (GFR)<60 ml/min/1.73 m^2^ and/or urinary albumin/creatinine ratio (ACR) >30 mg/g for more than three months [1]. The development of CKD is based on long-term latent impairment of renal function and progression of kidney damage, which can in a short time lead to the terminal stage of CKD. Because of this, the issue of early diagnosis of renal pathology and its prevention remains relevant.

One of the main methods of studying renal function is GFR, which can be determined in different ways. The most common method in the clinic is the calculation of GFR on the content of endogenous creatinine in serum. Previously methods for calculating GFR by inulin and ethylenediamine tetraacetic acid (EDTA) were widely used, identical to creatinine, filtered by the kidneys, not secreted and absorbed by the proximal and distal renal tubules [2]. Serum creatinine level is fairly constant, although it is influenced by some extrarenal factors: gender, age, muscle mass, excess protein in the diet, weight gain, dehydration etc. The "blind zone" phenomenon is also noteworthy, characterized by a disproportionate increase in creatinine and a decrease in GFR to 40–90 ml/min/1.73 m^2^. It is in the "blind zone" that the beginning of renal pathology development can often be missed [3]. It is important to remember that the creatinine content increases after a decrease in GFR by 50% or more, so the question arose about a more accurate method of determining GFR.

Serum cystatin C is a highly sensitive and accurate marker of renal filtration function belonging to the cysteine protease inhibitor family. This non-glycosylated protein is synthesized by all nuclear cells, not secreted by the proximal tubules of the kidneys, freely filtered through the glomerular membrane, and completely metabolized in the kidneys [4].

Normally, serum cystatin C levels are due to constant rates of its synthesis and excretion from the body (mainly through the kidneys). In a pathology, its level increases. The more severe the pathology of the kidneys, the slower cystatin C is filtered, and the higher the levels are determined in the serum. That is, its level in the blood practically depends only on GFR; no metabolic processes affect it.

Determination of cystatin C level seems promising to assess the functional status of the kidneys and the rate of progression of kidney tissue damage in various kidney pathologies (kidney disease, diabetes, hypertension, kidney transplantation).

LADA is one of the heterogeneous types of diabetes which combines symptoms of T1DM and T2DM and is diagnosed by detecting antibodies to islet antigens in the blood with manifestations of the disease in adults older than 35 years [5]. According to the updated classification of the ADA (2021), LADA is classified as T1DM, which develops in adulthood and has a slowly progressive course [6].

There is sufficient information on changes in the content of cystatin C in patients with CKD, which is caused by DKD on the background of T1DM and T2DM, an early predictor of this complication [7]. However, there are currently no such data for patients with LADA. The current results regarding chronic complications of LADA are mainly related to macrovascular lesions. Data on the frequency and structure of microvascular complications in LADA, particularly DKD, are scarce and quite contradictory, and there is almost no information about the peculiarities of their course.

The aim of the study was to determine the role of renal damage markers in the diagnosis of early-stage renal disease in patients with latent autoimmune diabetes in adults.

## MATERIALS AND METHODS

84 patients with DM were examined. Inclusion criteria: confirmed diagnosis of DM and CKD I-IV stages, which is caused by DKD; the age of patients older than 18 years, written informed consent to participate in the study. Exclusion criteria: other causes of CKD, CKD stage V, patients on hemodialysis or peritoneal dialysis, kidney transplant patients, acute infectious, inflammatory, and surgical pathology, clinically significant heart and liver failure, severe and mental illness, malignancies, other uncompensated chronic illnesses, antibacterials, glucocorticoids, nonsteroidal anti-inflammatory drugs, radiopaque and other agents that may have affected renal function at least 8 weeks prior to enrollment, pregnancy, lactation.

The diagnosis of LADA was established according to the recommendations of the Immunology of Diabetes Society [8]. All patients were tested for two types of antibodies to islet antigens – glutamic acid decarboxylase antibodies (antiGAD) and IA-2 antibodies (IA-2A ab) by enzyme-linked immunosorbent assay using DiaMetra SLR kits. (Italy). Peculiarities of DKD were studied based on anamnesis data, clinical examination, GFR, albuminuria, ACR etc [9].

GFR was assessed using six formulas: 3 formulas taking into account blood creatinine levels: CKD-EPI (Chronic Kidney Disease Epidemiology Collaboration), MDRD GFR (Modification of Diet in Renal Disease Study), and Cockcroft-Gault (CG) equation; 2 formulas using cystatin C taking into account sex of the patient – GFR cys C and GFR CKD-EPI cys C (2012); and 1 formula considering the level of both substances – GFR CKD EPI creat-cys C (2021) with the establishment of GFR category and albuminuria [4, 10]. The category of albuminuria was determined using microalbuminuria (MAU) and ACR indicators in the urine.

Statistical processing of the data obtained was performed in TIBCO Statistica version 13.3.0 (StatSoft) and Microsoft Excel 2016 using the Wilcoxon-Mann-Whitney U-test. The results were considered statistically significant at p<0.05.

## RESULTS

84 patients with DM and CKD caused by DKD (mean age of patients – 45.9±1.46 years; men – 41, women – 43; diabetic history – 11.7±1.04 years) and 25 representatives of the control group were examined. Patients were divided into three groups. The first group included 43 people with LADA, the second group 21 patients with T1DM, and the third 20 patients with T2DM.

At the first stage of our study, we assessed the differences in GFR between patients with diabetes and DKD calculated by different formulas. As can be seen from Table 1, the GFR rate using the CKD-EPI formula in patients with LADA and DKD did not significantly differ from that of CKD-EPI cysC, slightly different from MDRD results (10.6% higher, respectively), but 21.9% lower compared to CG formula.

**Table 1:** Variations of glomerular filtration rate in patients with different types of diabetes mellitus in combination with diabetic kidney disease depending on the applied formulas.

Formula	Control group (n=25)	LADA (n=43)	T1DM (n=21)	T2DM (n=20)
**Estimated GFR values taking into account serum creatinine**				
**GFR CKD-EPI, ml/min/1.73 m^2^**	106.8±4.75	63.5±2.86	73.1±5.09	56.6±3.56
**GRF MDRD, ml/min/1.73 m^2^**	108.7±3.91 p>0.05	57.4±2.72 p<0.05	66.6±4.42 p<0.05	53.8±3.24 p>0.05
**GFR CG, ml/min/1.73 m^2^**	104.3±4.07 p>0.05	81.3±3.86 p<0.01	81.4±7.66 p>0.05	80.9±4.52 p<0.01
**Estimated GFR values taking into account serum cystatin C**				
**GFR CKD-EPI cysC (2012), ml/min/1.73 m^2^**	93.9±7.35 p>0.05	61.5±6.29 p<0.05	58.9±8.58 p>0.05	45.6±6.31 p<0.01
**GFR cysC, ml/min/1.73 m^2^**	114.0±11.63 p>0.05	56.9±4.06 p>0.05	69.5±9.09 p<0.05	57.9±7.69 p>0.05
**Estimated GFR values taking into account serum creatinine and cystatin C**				
**GRF CKD-EPI creat-cysC (2021), ml/min/1.73 m^2^**	97.0±3.52 p>0.05	65.8±4.47 p>0.05	67.3±3.93 p>0.05	55.3±6.93 p>0.05

p – the probability of changes in GFR relative to the formula GFR CKD-EPI.

In T1DM, the GFR estimated by CKD-EPI was higher than that calculated by CKD-EPI cysC, CKD-EPI creat-cysC, GFR cysC, and MDRD GRF by 24.1%, 8.6%, 5.5%, and 9.8%, respectively, and lower with 10.2% relative to CG.

In the group of patients with T2DM, the GFR estimated by CKD-EPI was 24.1% higher than that calculated by the CKD-EPI cysC formula, which differed from the GFR calculated using the CKD-EPI creat-cysC, GFR cysC, and MDRD formulas with 5%, but lower than CG by 30.1%.

Consequently, using formulas based on the level of cystatin C allows a more objective assessment of the category of GFR and detection of renal dysfunction in the early stages of development.

**Figure 1:**
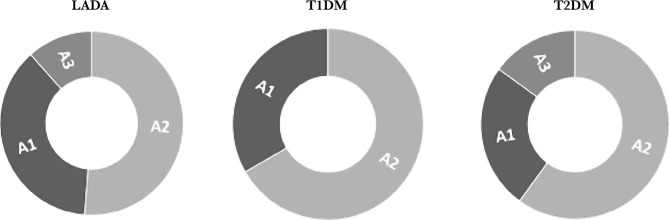
Distribution of patients with different types of diabetes by categories of albuminuria.

The next step in our study was to establish the features of albuminuria in patients with LADA compared with classical types of diabetes. Patients in all groups were dominated by microalbuminuria (Figure 1), which corresponds to the category of albuminuria A2 (51.2% of patients with LADA, 66.7% – with T1DM and 60.0% – with T2DM) and A1 (normoalbuminuria, in about one-third of patients with LADA and T1DM and 25% of patients with T2DM). It should be noted that category A3 (proteinuria) was quite rare (11.6% in LADA and 15% in T2DM groups).

The results of the albuminuria assessment in different LADA phenotypes were as follows: in LADA1, albuminuria category A1 was registered in 40% of patients, A2 – in 55%, and A3 – only in 5%, while in the LADA2 group, A1 was registered in 34.8%, A2 – 56.5%, and A3 – 8.7% of patients.

Depending on the presence of albuminuria, renal function significantly changed, and the level of creatinine and albumin in urine increased, and, as a consequence, the albumin-creatinine ratio (Table 2).

**Table 2:** Relationship between the degree of albuminuria in patients with diabetes mellitus and diabetic kidney disease with markers of renal damage and indicators of renal function.

Distributive feature	LADA, n=43		T1DM, n=21		T2DM, n=20	
	Category of albuminuria		Category of albuminuria		Category of albuminuria	
	A1 (n=16)	A2-3 (n=27)	A1 (n=7)	A2-3 (n=14)	A1 (n=5)	A2-3 (n=15)
**Serum creatinine, µmol/L** [11]	121.2±4.65	109.9±6.95 p_1_<0.05	121.7±7.42	93.1±4.43 p_2_<0.01	107.3±3.51	116.2±10.18 p_3_<0.05
**Cystatin C, mg/L**	1.74±0.202	1.34±0.120 p_1_>0.05	1.20±0.200	1.58±0.217 p_2_<0.01	1.60±0.261	1.75±0.215 p_3_<0.05
**GFR CKD-EPI, ml/min/1.73 m^2^**	58.4±1.01	66.5±4.45 p_1_>0.05	49.9±3.41	84.7±5.13 p_2_<0.01	56.6±1.64	56.6±4.77 p_3_>0.05
**GFR CKD-EPI cysC (2012), ml/min/1.73 m^2^**	52.0±7.79	67.4±8.88 p_1_<0.01	62.0±16.00	58.3±10.10 p_2_<0.01	46.4±8.83	45.1±9.11 p_3_<0.01
**GRF CKD-EPI creat-cysC (2021), ml/min/1.73 m^2^**	58.3±5.32	70.0±6.23 p_1_>0.05	58.0±4.00	69.2±4.47 p_2_<0.01	64.0±12.00	51.0±8.61 p_3_>0.05
**Urine albumin, mg**	11.0±1.03	322.5±87.83 p_1_<0.01	11.4±1.04	144.6±19.91 p_2_<0.01	17.2±1.07	340.0±95.09 p_3_>0.05
**Urine creatinine, mmol/L**	18.5±2.70	25.2±2.73 p_1_<0.05	18.7±4.71	31.6±12.77 p_2_>0.05	10.8±2.06	12.5±1.95 p_3_<0.05
**Albumine-creatinine ratio, mg/mmol**	0.74±0.107	25.15±2.732 p_1_<0.01	0.79±0.154	12.01±3.528 p_2_<0.01	1.80±0.270	23.53±3.767 p_3_<0.01

p_1_ – probability when comparing the group of normoalbuminuria with respect to the group of albuminuria in LADA; p_2_ – probability when comparing the group of normoalbuminuria relative to the group of albuminuria in T1DM; p_3_ – probability when comparing the group of normoalbuminuria with respect to the group of albuminuria in T2DM.

In particular, in patients with LADA, the content of albumin in the urine in the group with normoalbuminuria (A-) probably differed 29.3 times from that in patients with albuminuria (A+) (p<0.01), in T1DM – in 12.6 times (p<0,01), and in T2DM changes in this indicator were insignificant. The level of creatinine in urine increased by 35.9% (p<0.05) in A+ against the group with A- in LADA, in the group of T2DM – by 15.5% (p<0.05) and did not undergo statistically significant changes in T1DM. ACR in the LADA group was 34.0 times higher in A+ compared with A- (p<0.01), 15.2 times in T1DM (p<0.01), and 13.1 times in T2DM (p<0.01). The level of creatinine in the blood decreased by 9.3% and 23.5% in A+ compared with group A- in patients with LADA (p<0.05) and T1DM (p<0.01), respectively, while it was probably 0.9% lower in patients with T2DM and A- *versus* the group with T2DM and A+. Cystatin C and GFR did not change significantly according to all formulas in LADA (p^˃^0.05). In contrast, in patients with T1DM and T2DM, the level of cystatin C significantly increased together with the degree of albuminuria by 31.7% (p<0.01) and 9.4% (p<0.05), respectively.

GFR underwent significant changes in all formulas only in patients with T1DM. Thus, according to the CKD-EPI formula, GFR was 41.0% lower in group A- than in A+ (p<0.01), when recalculated according to CKD-EPI cysC – 6.3% higher (p<0.01), and according to the CKD-EPI creat-cysC formula – 16.2% lower than with A+ (p<0.01). In the LADA and T1DM groups, the difference was observed only when using the CKD-EPI cysC formula – GFR in LADA increased by 29.3% in group A+ compared with A- (p<0.01), and in T2DM, it decreased by 2.7% (p<0.01).

GFR correlations were revealed according to the CKD-EPI formula with the level of cystatin C (r=-0.5, p=0.001), serum potassium (r=-0.9, p=0.000), serum creatinine level (r=-0.8, p=0.000), urea level (r=-0.3, p=0.030) and urine creatinine (r=-0.3, p=0.048), according to the CKD-EPI cysC formula – with cystatin C (r=-0.7, p=0.000), glycated hemoglobin (r=0.3, p=0.047), serum creatinine level (r=-0.4, p=0.035), according to the CKD-EPI creat-cysC formula – with the level of cystatin C (r=-0.7, p=0.000), glycated hemoglobin (r=0.3, p=0.049), serum potassium (r=-0.7, p=0.001), serum creatinine (r=-0.4, p=0.029) and urinary creatinine (r=-0.4, p=0.034). At the same time, no probable associations with indicators of autoimmune load, insulin resistance, degree of albuminuria were established.

## DISCUSSION

Determining GFR using creatinine is not always sufficient for an accurate diagnosis of renal function, especially in the early stages of CKD. In contrast, cystatin C is a highly sensitive and accurate endogenous marker of GFR levels in patients with latent renal tissue damage compared to the traditional marker creatinine, which is associated with a constant rate of synthesis and glomerular secretion, as evidenced by our study.

The closest to the recommended CKD-EPI formula was GFR, calculated taking into account the rate of cystatin C as an early marker of kidney damage. In patients with LADA and T1DM, GFR was higher in cases with existing albuminuria, regardless of the formulas used. Thus, the nonalbuminuria phenotype, which, according to our study, occurs in 37.2% of patients with LADA, is accompanied by a greater degree of renal impairment, which indicates the need to determine serum cystatin C in the early stages of LADA.

The correlation analysis showed a strong strength between serum creatinine and GFR, calculated according to the formula of creatinine only and medium strength – with cystatin C, while in the case of GFR formula by cystatin and creatinine, there was an inversely proportional relationship: strong strength with cystatin C and medium strength with serum creatinine. Interestingly, medium-strength inverse correlations with serum creatinine were observed when we calculated GFR using cystatin C only, which supports the independence and authenticity of this formula.

No association was established between albuminuria and GFR, as well as cystatin C levels in patients with LADA, suggesting that GFR calculated by cystatin C formula can be considered an independent marker for assessing renal impairment in this type of diabetes, especially in patients with non albuminuric DKD phenotype.

## CONCLUSION

Serum cystatin C levels are the most accurate, early, and independent predictors of the development and progression of chronic kidney disease in patients with diabetes mellitus, including latent autoimmune diabetes in adults. Determining this marker and assessing albuminuria can help stop the development of kidney damage, prolong the life expectancy of patients with diabetes and improve its quality.
